# Correlation Between Glycemic Biomarkers and Continuous Glucose Monitoring Metrics in Chronic Kidney Disease: A Systematic Review and Correlation Meta-Analysis

**DOI:** 10.3390/jcm15166137

**Published:** 2026-08-07

**Authors:** Miguel Angel Cuevas-Budhart, Karen Gómez-Rosas, Rubén David Saavedra-Fernández, Marcela Ávila-Díaz, Erwin Campos, Aldo Ferreira-Hermosillo, Alfonso Ramos-Sánchez, Iván Cavero-Redondo, Ramón Paniagua

**Affiliations:** 1 Unidad de Investigación Médica en Enfermedades Nefrológicas CMN Siglo XXI, Instituto Mexicano del Seguro Social, Mexico City 06600, Mexico; angel_budhart@hotmail.com (M.A.C.-B.); cramav@gmail.com (M.Á.-D.); 2 Unidad Académica Escuela Nacional de Medicina y Homeopatía, Instituto Politécnico Nacional, Mexico City 07320, Mexico; karengomras@gmail.com (K.G.-R.); savedrafer1952@gmail.com (R.D.S.-F.); 3 Macrotech, Santo Domingo 10902, Dominican Republic; erwinivan@hotmail.com (E.C.); alfonso.ramo@gmail.com (A.R.-S.); 4 Unidad de Investigación Médica en Enfermedades Endocrinológicas CMN Siglo XXI, Instituto Mexicano del Seguro Social, Mexico City 06600, Mexico; aferreira@endocrinologia.org.mx; 5 CarVasCare Research Group, Instituto de Biomedicina y Facultad de Enfermería de Cuenca, Universidad de Castilla-La Mancha, 16002 Cuenca, Spain; ivan.cavero@uclm.es

**Keywords:** chronic kidney disease, diabetes, glycated hemoglobin, continuous glucose monitoring, meta-analysis

## Abstract

**Background/Objectives:** Accurate assessment of glycemic control in patients with diabetes and chronic kidney disease (CKD) is challenging because alterations associated with CKD affect the performance of conventional glycemic biomarkers, such as glycated hemoglobin (HbA1c), glycated albumin (GA), and fructosamine, while continuous glucose monitoring (CGM) offers a more comprehensive assessment of glycemic exposure; however, a quantitative synthesis of the correlation between these biomarkers and CGM-derived metrics across CKD stages is lacking. To systematically evaluate the correlation between HbA1c, GA, and fructosamine and CGM-derived glycemic metrics in adults with diabetes and CKD, using CGM as the reference standard. **Methods:** A systematic review with correlation meta-analysis was conducted following the Preferred Reporting Items for Systematic Reviews and Meta-analyses (PRISMA) guidelines, including studies published between January 2016 and March 2025, indexed in PubMed, Scopus, and Web of Science. Correlation coefficients were transformed using Fisher’s z transformation and pooled using a random-effects model; heterogeneity was assessed using the I^2^ statistic. **Results:** Ten studies met the eligibility criteria; six provided sufficient data for meta-analysis. The pooled correlation was moderate (r = 0.66; 95% confidence interval [CI]: 0.56–0.74) with substantial heterogeneity (I^2^ = 58.9%). In exploratory biomarker-specific subgroup analyses, GA showed the numerically highest correlation with CGM-derived metrics (r = 0.74; 95% CI: 0.65–0.81), followed by HbA1c (r = 0.65; 95% CI: 0.51–0.75); fructosamine was more variable. **Conclusions:** No single biomarker demonstrated consistent superiority across CKD stages and treatment modalities. These correlation analyses measure linear association, not clinical interchangeability. Biomarker selection should be individualized according to CKD stage, dialysis status, and CGM availability.

## 1. Introduction

Diabetes is among the leading causes of morbidity worldwide. According to the 11th edition of the International Diabetes Federation Diabetes Atlas, an estimated 589 million adults aged 20–79 years were living with diabetes in 2024, corresponding to a global prevalence of 11.1%, with projections approaching 853 million by 2050 [1]. Chronic kidney disease (CKD) is closely linked to this trend: diabetes is currently estimated to account for approximately 36% of the global CKD burden, and both conditions are expected to continue rising in parallel over the coming decades [2]. Diabetes-related CKD is also associated with a disproportionate share of disease-related mortality and disability-adjusted life-years, particularly in low- and middle-income regions [3]. Diabetes is the leading cause of CKD and progression to end-stage kidney disease, making the accurate assessment of glycemic control in this population critically important. Glycated hemoglobin (HbA1c) has historically been considered the reference standard for estimating chronic glycemic exposure; however, its validity in patients with CKD, particularly in advanced stages, is compromised by several factors including anemia, reduced red blood cell lifespan, the use of erythropoiesis-stimulating agents, and processes such as carbamylation, all of which alter the glycation kinetics and introduce systematic bias into its interpretation [1,2,3].

Several studies have demonstrated that the correlation between HbA1c and actual glycemia declines as CKD progresses [4]. In advanced stages of CKD, the association between HbA1c with metrics derived from continuous glucose monitoring (CGM) becomes significantly attenuated and may even disappear [5]. In patients undergoing hemodialysis, substantial discordance has been reported between HbA1c and the glycemic management index (GMI), with differences exceeding 1% in a considerable proportion of cases [6], and clinically significant discrepancies observed in more than 60% of patients with CKD [7].

Given these limitations, alternative biomarkers such as glycated albumin (GA) and fructosamine have been proposed. GA has demonstrated a stronger association with plasma glucose in patients receiving dialysis and may have prognostic value in advanced CKD [8,9,10], however, its interpretation may also be influenced by factors such as nutritional status and body composition [11]. Fructosamine, in contrast, reflects short-term glycemic control, although its comparative performance has been inconsistent and has not demonstrated clear superiority over HbA1c [12,13,14].

Importantly, CGM-based studies have shown that none of these biomarkers adequately captures glycemic variability or hypoglycemic episodes, both of which are clinically relevant in patients with CKD [15,16,17,18,19]. Consequently, uncertainty remains regarding the relative performance of HbA1c, GA, and fructosamine for estimating glycemic control in patients with CKD, particularly when compared with CGM-derived metrics.

Although several primary studies have evaluated the relationship between HbA1c, glycated albumin, fructosamine, and continuous glucose monitoring in patients with CKD, their findings have been inconsistent because of differences in CKD stage, dialysis modality, reference standards, and study design. To date, no systematic review has quantitatively synthesized these correlations while specifically prioritizing CGM-derived metrics as the reference standard. Consequently, the comparative performance of these biomarkers remains uncertain, limiting evidence-based recommendations for glycemic assessment in CKD.

Therefore, the aim of this study was to systematically and quantitatively evaluate the association and comparative performance of these biomarkers through a correlation meta-analysis with mean glucose, CGM-derived metrics, and other reference standards, as well as identify clinical factors that may modify these relationships in patients with diabetes and CKD.

The primary objective of this review was to quantify, through correlation meta-analysis, the strength of association between HbA1c, GA, and fructosamine and CGM-derived metrics in adults with diabetes and CKD (Research Question 1: What is the pooled correlation between glycated biomarkers and CGM-derived metrics in patients with CKD?). The secondary objectives were: (1) to compare the magnitude of these correlations across CKD severity categories and dialysis modalities (Research Question 2: Does this correlation vary according to CKD stage or dialysis modality?); and (2) to identify clinical and methodological factors associated with heterogeneity in reported correlations (Research Question 3: Which clinical or methodological factors moderate the magnitude of this association?). All secondary analyses were pre-specified as exploratory given the limited number of available studies (k = 6 for quantitative synthesis), ensuring alignment between the stated objectives, methods, and the exploratory framing applied in Section 3.7, Section 3.8 and Section 3.9.

Despite individual reports on biomarker limitations in CKD, there is a lack of a standardized, quantitative synthesis that compares the relative accuracy of HbA1c, GA, and fructosamine against CGM metrics across the full spectrum of renal dysfunction.

To date, no systematic review has quantitatively synthesized correlations between HbA1c, GA, and fructosamine and CGM-derived metrics while specifically restricting the primary analysis to studies using CGM as the reference standard. Prior narrative reviews [5,9,20] summarized individual study findings but did not apply a pre-specified hierarchical scheme for CGM metric selection or explicitly separate CGM-referenced from non-CGM-referenced comparisons at the pooling stage. The present study addresses this gap by providing the first correlation meta-analysis that: (1) restricts the primary quantitative synthesis to CGM-referenced studies; (2) applies a pre-specified hierarchical selection scheme (mean CGM glucose > GMI > TIR > FPG); and (3) quantifies stage-dependent correlation attenuation across the full CKD spectrum. These findings are intended to inform clinicians on when conventional biomarkers remain adequate and when CGM or alternative markers should be prioritized in clinical practice.

This study addresses this gap by providing a meta-analytical framework to determine when these biomarkers remain clinically reliable, thereby informing evidence-based decisions in nephrological care.

## 2. Materials and Methods

### 2.1. Study Design

This study employed a correlation meta-analysis framework with a pre-registered protocol (PROSPERO ID: 1375033). The PRISMA 2020 statement was used exclusively as a transparency reporting guideline [21]; all methodological decisions, including eligibility criteria, effect measure selection, evidence hierarchy, and synthesis approach, were determined a priori based on the research questions and the analytical requirements of correlation meta-analysis, independently of the PRISMA checklist structure (Supplementary S1). Correlation meta-analysis was selected because the primary outcome of interest was the strength of association between glycemic biomarkers and CGM-derived metrics rather than diagnostic accuracy or treatment effect.

### 2.2. Eligibility Criteria

Studies were included if they met the following criteria: original studies in adult populations with diabetes and CKD; assessment of at least one of the glycation biomarkers of interest (HbA1c, GA or fructosamine); comparison of these markers with reference measures of glycemic exposure, including mean glucose, continuous glucose monitoring, or plasma glucose; and reporting quantitative associations or clinical outcomes. Only articles published in English or Spanish were considered.

Studies were excluded if they involved pediatric populations, were editorials, letters to the editor, commentaries, narrative reviews, or abstracts without full text; if they were animal experimental studies; if they lacked specific data in patients with chronic kidney disease; or if they were duplicate reports.

### 2.3. Information Sources and Search Period

A systematic literature search was conducted in the PubMed, Scopus, and Web of Science databases to identify studies evaluating glycation biomarkers in patients with diabetes and CKD.

The search included studies published between 1 January 2016 and March 2025, with no geographic restriction. Studies published in English and Spanish were included. The search strategy was developed using a combination of controlled vocabulary (MeSH terms) and free-text keywords, applying the Boolean operators AND and OR to maximize sensitivity and specificity (see Supplementary Materials Table S1).

Semi-automated title and abstract screening was performed using Rayyan (Rayyan Systems Inc., Doha, Qatar, web version), a web-based systematic review platform. Rayyan was configured to flag records meeting predefined exclusion criteria (pediatric populations, non-CKD populations, non-human studies, reviews, editorials, and conference abstracts without full text). Records automatically flagged were reviewed by at least one author before exclusion to confirm appropriateness. All final inclusion and exclusion decisions at the full-text stage were made independently by two reviewers without automated assistance.

### 2.4. Search Strategy

The search strategy was developed using a combination of controlled vocabulary and free-text terms, structured into three conceptual domains: glycation biomarkers, chronic kidney disease, and glycemic control. The Boolean operators AND and OR were applied to maximize search sensitivity while maintaining specificity.

The main concepts included:

- Glycation biomarkers
  HbA1c, GA, fructosamine
- Clinical condition
  Chronic kidney disease, CKD, renal insufficiency, renal failure, dialysis
- Outcome or construct of interest
  Glycemic control, metabolic control, accuracy, validity, reliability, correlation, continuous glucose monitoring

The specific search strategies were adapted to each database and are provided in the Supplementary Materials. In addition, a manual search of the reference lists of included studies was performed.

### 2.5. Study Selection Process

Two independent reviewers screened the titles and abstracts, followed by full-text assessment of potentially eligible studies. Discrepancies were resolved through consensus. The selection process was documented using a PRISMA flow diagram [21].

### 2.6. Data Extraction

Two reviewers independently extracted data using a piloted standardized form. Disagreements were resolved by consensus. The following variables were collected: author, year of publication, country, study design, sample size, population characteristics, stage of chronic kidney disease, treatment modality (dialysis or non-dialysis), biomarkers assessed, reference method, and quantitative outcomes.

CGM-derived metrics were prioritized when available due to their greater physiological relevance for estimating glycemic exposure.

### 2.7. Effect Measures

The primary outcome was the correlation coefficient between glycation biomarkers (HbA1c, GA and fructosamine) and reference metrics of glycemic exposure.

Correlation coefficients reported in the primary studies were considered the primary outcome measure and were transformed using Fisher’s z transformation for quantitative synthesis.

To ensure methodological rigor and minimize selection bias, we implemented a scientific standardization protocol for estimate selection. The unit of analysis was the effect size derived from each study. When multiple estimates were reported within a single study, a single representative estimate was selected using predefined hierarchical criteria to preserve statistical independence. This hierarchy prioritized metrics based on their physiological validity for capturing chronic glycemic exposure, in the following order: (1) CGM-derived metrics (mean CGM glucose > glucose management indicator [GMI] > time in range [TIR]) > fasting plasma glucose (only when no CGM-derived metric was available); (2) larger sample size; and (3) greater clinical relevance of the outcome.

### 2.8. Statistical Analysis

Correlation coefficients reported in primary studies were transformed using Fisher’s z transformation to stabilize variance and approximate a normal distribution of effect sizes. The variance of each estimate was calculated based on the sample size of each study.

Transformed size effects were pooled using a DerSimonian and Laird random-effects model due to anticipated clinical and methodological heterogeneity across populations, biomarkers assessed, and reference methods used.

Between-study heterogeneity was assessed using Cochran’s Q statistic and quantified using the I^2^ index.

When a study reported multiple glycemic metrics, the measure with the highest physiological validity was prioritized as the reference, following a predefined hierarchical order: mean glucose derived from continuous glucose monitoring, glucose management indicator, time in range, and fasting plasma glucose.

The main meta-analysis was restricted to studies that used continuous glucose monitoring as the reference standard. Studies based on fasting plasma glucose were included only in the narrative synthesis to minimize bias related to methodological comparability.

Statistical analyses were performed in the R environment using the metafor and meta packages.

### 2.9. Stratification and Subgroup Analysis

A priori stratified analyses were conducted to explore sources of clinical and methodological heterogeneity. The analyses were structured according to:

- Biomarker type: HbA1c, GA and fructosamine.
- Glycemic reference method: continuous glucose monitoring, including derived metrics such as mean glucose, glucose management indicator, and time in range, as well as fasting plasma glucose or estimated glucose metrics.

In addition, subgroup analyses were performed according to relevant clinical characteristics, including stage of chronic kidney disease when reported, and renal replacement therapy modality, distinguishing between dialysis and non-dialysis patients.

When available, additional exploratory analyses were conducted based on potential effect modifiers such as population type, sample size, and study design characteristics.

### 2.10. Heterogeneity Assessment

Between-study heterogeneity was evaluated using the I^2^ statistic, derived from Cochran’s Q test, which quantifies the proportion of total variability attributable to between-study heterogeneity rather than sampling error. The following thresholds were used for interpretation: values below 25% indicated low heterogeneity, values between 25% and 50% indicated moderate heterogeneity, and values above 50% indicated high heterogeneity.

### 2.11. Sensitivity Analysis

Sensitivity analyses were performed using leave-one-out analyses and the sequential exclusion of studies at high risk of bias to assess the stability of the results.

### 2.12. Synthesis of Secondary Outcomes

Due to heterogeneity in reporting, secondary outcomes, including area under the curve, odds ratio, and hazard ratio, were synthesized narratively without quantitative pooling.

### 2.13. Publication Bias

Publication bias was assessed through visual inspection of funnel plots constructed from transformed correlation coefficients. In addition, when the number of included studies allowed, statistical tests for funnel plot asymmetry were considered. However, due to the limited number of studies included in the meta-analysis, the power to detect asymmetry was reduced, and the results were interpreted with caution.

### 2.14. Methodological Quality and Risk of Bias

The methodological quality of all 10 included studies was assessed using the Newcastle–Ottawa Scale (NOS) adapted for cross-sectional and cohort observational designs. The NOS evaluates three domains: selection of study groups, comparability of groups, and assessment of outcome or exposure. Each study was scored independently by two reviewers, with discrepancies resolved by consensus. Studies scoring 7 or above were classified as high quality; scores of 4–6 indicated moderate quality; scores below 4 indicated low quality. The NOS was selected as the primary quality assessment tool because it is better suited to the observational correlation designs represented in this review. The protocol for this systematic review was registered in PROSPERO (ID: 1375033) prior to study selection and data extraction. The adoption of the NOS as the primary quality assessment instrument, in place of the QUADAS-2 specified in the registered protocol, represents a post hoc protocol deviation; this change was made because NOS is more appropriate for the predominantly observational correlation designs of the included studies, and is acknowledged as a deviation here.

### 2.15. Ethical Considerations

This study was conducted as a systematic review based on previously published data; therefore, no direct participation of human subjects or handling of identifiable information was involved. Accordingly, ethical committee approval was not required.

The protocol was registered in the international PROSPERO database (ID: 1375033), ensuring methodological transparency and reducing the risk of unnecessary duplication.

## 3. Results

### 3.1. Study Selection

The systematic search identified 1136 records across electronic databases, including PubMed (*n* = 49), Scopus (*n* = 612), and Web of Science (*n* = 475). Before screening, 23 duplicate records were removed, together with 295 records excluded through automated screening tools for not meeting the eligibility criteria and 25 additional records excluded for other reasons, resulting in 793 records for title and abstract screening.

During the screening phase, 762 records were excluded for failing to meet the inclusion criteria, and 31 studies were selected for full-text review. Of these, 7 reports could not be retrieved, leaving 24 studies for eligibility assessment. Following full-text evaluation, 14 studies were excluded for the following reasons: assessment of unrelated biomarkers (*n* = 5), involved non-relevant therapeutic designs (*n* = 3), and limited applicability of the study objective (*n* = 6).

**Figure 1:**
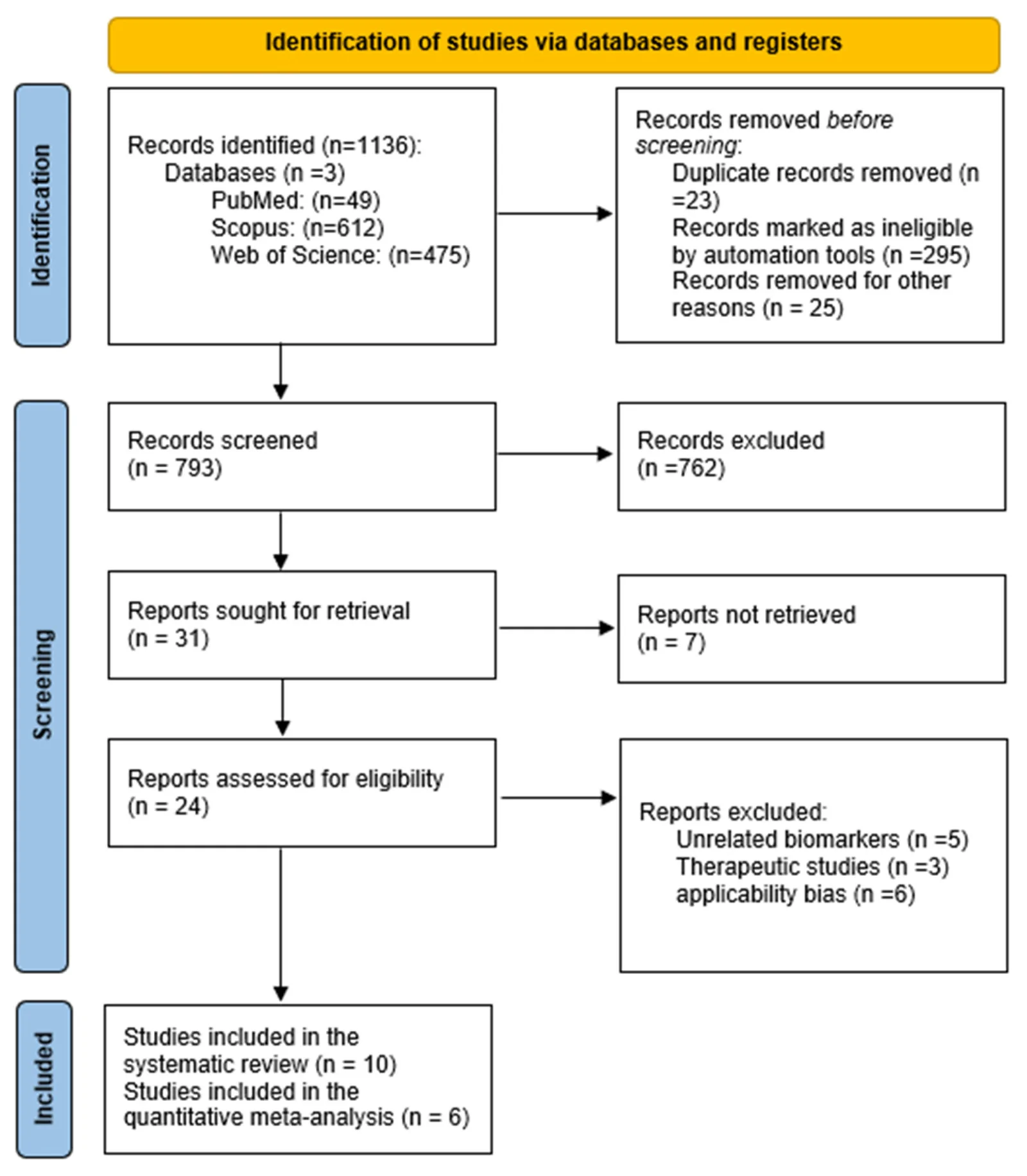
PRISMA 2020 flow diagram.

Finally, 10 studies were included in the systematic review, of which 6 provided sufficient data for inclusion in the quantitative meta-analysis (Figure 1).

### 3.2. Study Characteristics

Ten original studies were included, predominantly observational in design, comprising cross-sectional studies, prospective cohorts, and studies incorporating CGM. The study populations included patients with diabetes and CKD across different stages, ranging from non-dialysis CKD to end-stage kidney disease requiring hemodialysis or peritoneal dialysis. Sample sizes were heterogeneous, ranging from small CGM-based cohorts to large population-based studies [8,9,10,11,15,18,22,23,24].

The most frequently evaluated biomarkers were HbA1c, GA and fructosamine. Some studies performed direct comparisons among these biomarkers, whereas others assessed their correlation with plasma glucose or their agreement with CGM-derived metrics [8,12,15,19,22,23].

Reference measures of glycemic exposure were heterogeneous across studies. In several studies, CGM was used to estimate mean glucose, glucose management indicator, time in range, time above range, and glycemic variability, whereas other studies used fasting or postprandial plasma glucose as glycemic comparators.

For the purposes of this review, studies were classified into two analytical tiers: (1) a primary quantitative synthesis restricted to studies using CGM-derived metrics as the reference standard [7,8,9,17,22,23] and (2) a narrative synthesis including studies using fasting or postprandial plasma glucose as the comparator [11,15,16,25]. Studies in the second tier are not included in any pooled analyses and are clearly labeled throughout Section 3. The main characteristics of all included studies are summarized in Table 1.

### 3.3. Methodological Quality and Risk of Bias

**Figure 2:**
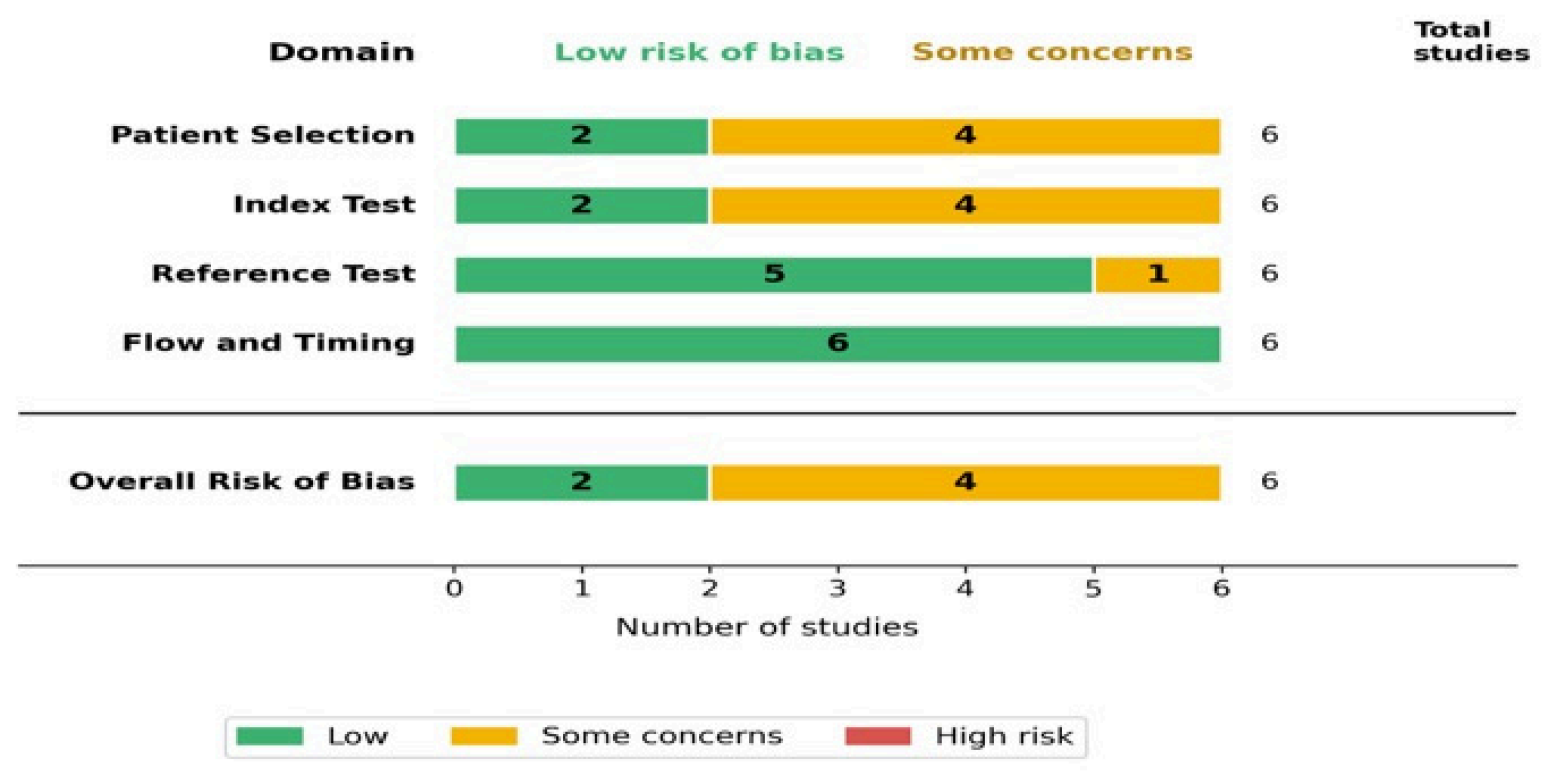
Risk of bias assessment. Newcastle–Ottawa Scale (NOS) applied to all 10 included studies. QUADAS-2 supplementary results for CGM-referenced studies are presented in Supplementary Table S3.

Methodological quality was assessed for all 10 included studies using the Newcastle–Ottawa Scale (NOS). Six studies were rated as high quality (NOS score 7–9): Galindo et al. [9], Ling et al. [8], Zelnick et al. [17], Kaminski et al. [22], Oriot et al. [10], and Ushigome et al. [23]. Four studies received moderate-to-high risk ratings: Kobayashi et al. [11], Ushigome et al. [23], Ha et al. [15], and Kaminski et al. [22] (NOS score 4–6, reflecting the small, highly selected sample in the burnt-out diabetes cohort). No study was rated as low quality overall. The most common sources of methodological concern were small sample sizes (Kobayashi et al. [11], *n* = 40; Ushigome et al. [23], *n* = 18; and Kaminski et al. [22], *n* = 40), single-center recruitment limiting external validity, and absence of blinding procedures in biomarker assessment. Full domain-level NOS scores for all 10 studies are presented in Supplementary Table S3. For the six CGM-referenced studies, QUADAS-2 was additionally applied given the diagnostic accuracy structure of those comparisons; results are presented in Supplementary Table S2. Updated Figure 2 presents the QUADAS-2 risk-of-bias assessment, summarized by domain, for the six Tier 1 studies; the corresponding NOS-based assessment for all 10 studies is presented in Supplementary Table S3.

Overall, methodological quality was considered acceptable; however, heterogeneity in study design, CKD populations, and glycemic assessment methods limited direct comparability across studies.

### 3.4. Individual Study Findings

The included studies evaluated the performance of glycemic biomarkers against different reference standards, primarily CGM and plasma glucose [8,9,18].

In studies using CGM, HbA1c demonstrated a progressive decline in concordance with mean glucose as CKD advanced, with reported discrepancies relative to the GMI and other CGM-derived metrics [8,9,10].

Regarding GA, several studies conducted in patients receiving dialysis reported stronger associations with plasma glucose and clinically relevant differences compared with HbA1c. However, in non-dialysis CKD populations, findings were inconsistent, with evidence suggesting susceptibility to non-glycemic influences [11,16,18,23].

Fructosamine demonstrated heterogeneous performance across studies, with correlations generally comparable to or lower than HbA1c, and without consistent evidence of superiority [15,18].

Overall, the individual study findings indicate substantial variability in biomarker performance, influenced by the CKD stage, treatment modality, and the reference method employed (see Supplementary Materials Table S2).

### 3.5. Synthesis of Results

Across the included studies, biomarker performance varied according to CKD severity, dialysis status, and the glycemic reference method used.

Studies using CGM consistently showed attenuation of the relationship between HbA1c and CGM-derived metrics as kidney function declined [8,9,10,23]. In advanced CKD and patients undergoing dialysis, HbA1c showed substantial discordance with glucose management indicator and mean CGM glucose, suggesting underestimation of glycemic exposure [8,9,10,23].

GA demonstrated stronger associations with plasma glucose and CGM-derived metrics in patients undergoing dialysis, particularly in peritoneal dialysis, where it outperformed HbA1c as a marker of glycemic exposure [11,23]. However, in non-dialysis CKD populations, its performance was inconsistent and appeared to be influenced by non-glycemic factors [16,18,22].

In contrast, fructosamine demonstrated heterogeneous performance across studies, with correlations generally comparable to or lower than HbA1c and without consistent evidence of superiority [15,16,18].

Overall, the included studies suggest that the validity of glycemic biomarkers in CKD is highly context-dependent and influenced by kidney disease severity, dialysis modality, and the glycemic reference method used. CGM emerged as the most consistent approach for estimating glycemic exposure and identifying limitations of conventional biomarkers [8,9,10,18,23].

### 3.6. Quantitative Synthesis of Correlations

The primary quantitative synthesis was restricted to the six studies using CGM-derived metrics as the reference standard Galindo et al. [9], Ling et al. [8], Zelnick et al. [17], Kaminski et al. [22], Oriot et al. [10], and Ushigome et al. [23], consistent with the pre-specified analytical plan. Studies using fasting or postprandial plasma glucose as the comparator were excluded from all pooled analyses and are described in the narrative synthesis only. For each included study, a single representative effect estimate was selected according to the hierarchical rule described in Section 2.7. A complete extraction table documenting all eligible correlations, the selected estimate, the reference metric, and the selection rationale for each study is provided in Supplementary Table S2. Six studies evaluating the correlation between glycemic biomarkers and CGM to derived metrics were included in the quantitative synthesis. The pooled analysis demonstrated a moderate overall correlation under a random-effects model (r = 0.66; 95% CI: 0.56–0.74), with substantial between-study heterogeneity.

Individual correlation coefficients ranged from r = 0.22 to r = 0.78. The highest correlation was observed in Zelnick et al. [17], whereas the lowest was reported in the CKD G5 subgroup of Ling et al. [8], where the association between HbA1c and CGM-derived metrics did not reach statistical significance.

**Figure 3:**
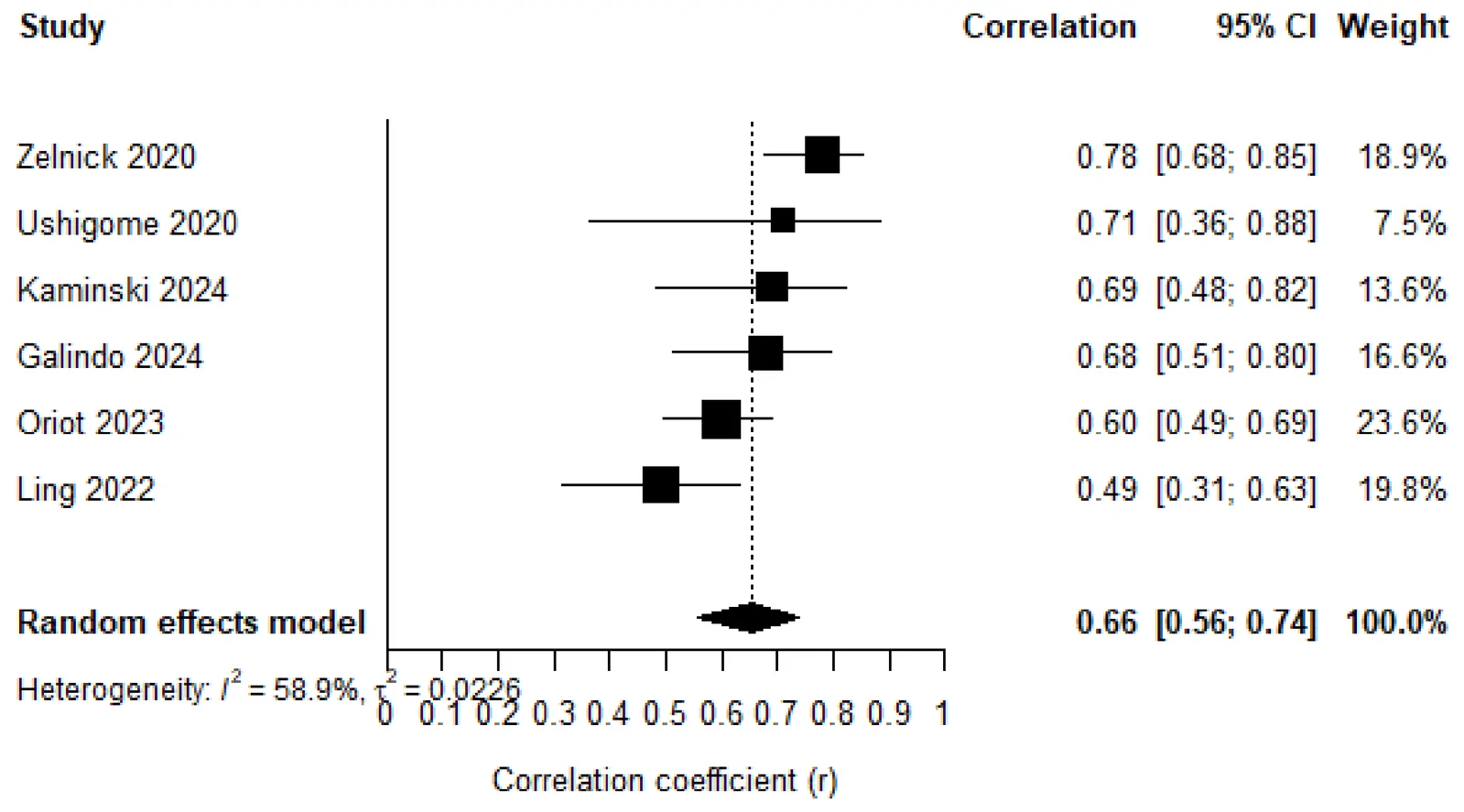
Forest plot of the correlation between glycemic biomarkers and continuous glucose monitoring (CGM)-derived metrics in patients with chronic kidney disease. The primary quantitative synthesis was restricted to six studies using CGM-derived metrics as the reference standard (Tier 1). Each square represents the point estimate of the correlation coefficient for an individual study, with horizontal lines indicating 95% confidence intervals. The size of the square reflects the relative weight of each study. The diamond represents the pooled correlation under a random-effects model (r = 0.66; 95% CI 0.56–0.74). The vertical dashed line indicates the null correlation (r = 0) [8,9,10,17,22,23].

A progressive attenuation in biomarker correlation was observed as renal dysfunction advanced. Correlations remained moderate in CKD G3b and G4 populations but declined markedly in advanced CKD and dialysis settings. The study-specific correlation coefficients and corresponding 95% confidence intervals are presented in Figure 3.

Additionally, four studies identified through the systematic search were not included in the primary quantitative synthesis because they did not directly evaluate correlations with CGM-derived metrics. These studies focused on secondary diagnostic utility, prognostic value or pathophysiological factors affecting biomarker interpretation, and were therefore incorporated only into the qualitative synthesis.

### 3.7. Biomarker-Specific Subgroup Analyses (Exploratory)

**Figure 4:**
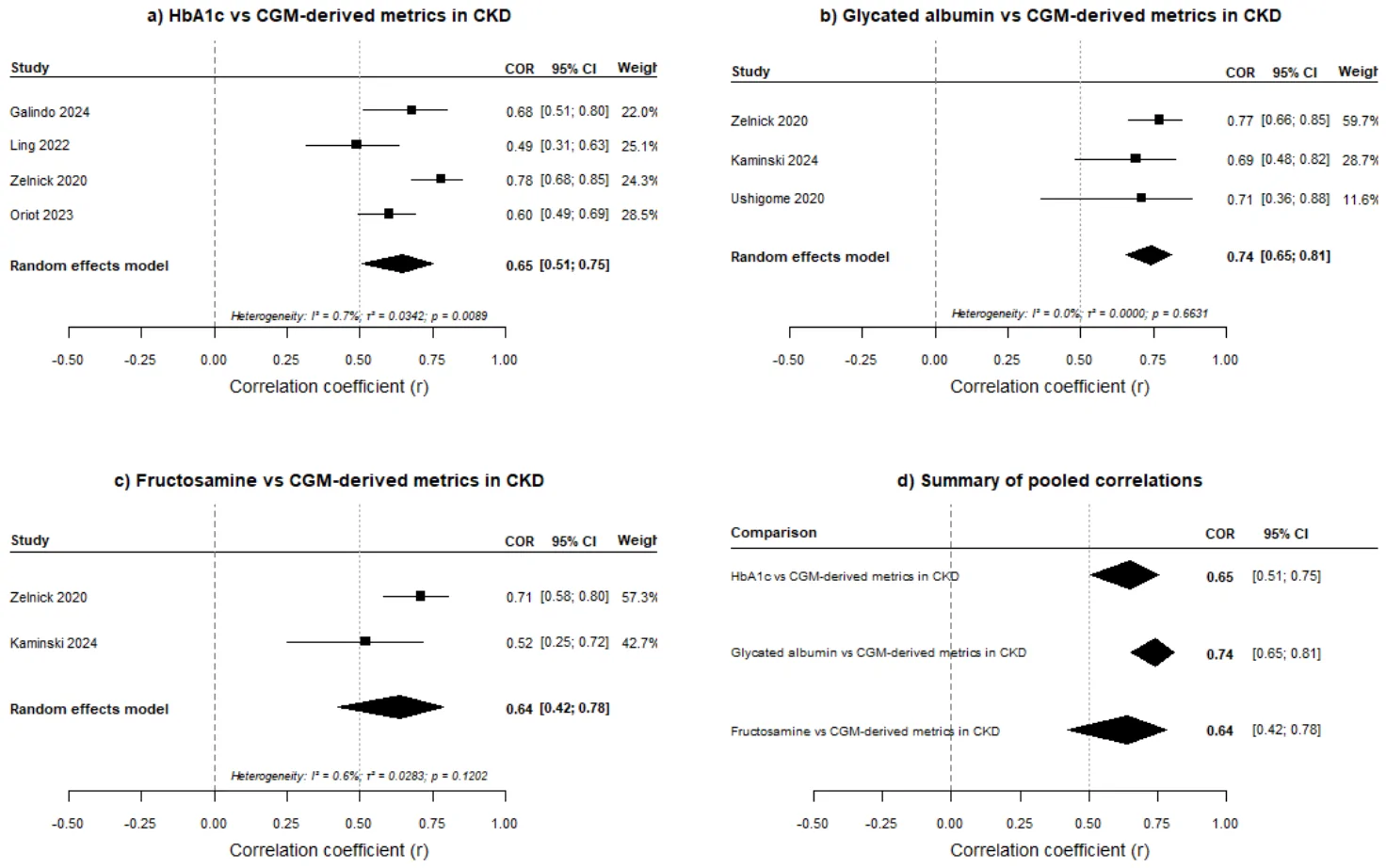
Subgroup analyses of correlations between glycemic biomarkers and continuous glucose monitoring (CGM)-derived metrics in patients with chronic kidney disease [8,9,10,17,22,23]. Panels (**a**–**c**) display forest plots for HbA1c, glycated albumin, and fructosamine, respectively. Correlation coefficients were pooled using random-effects models after Fisher z transformation and are presented as retransformed correlation coefficients (r) with 95% confidence intervals. Squares represent individual study correlation coefficients, with their size proportional to the relative study weight, and diamonds represent pooled estimates derived from random-effects models. Panel (**d**) provides a summary of pooled correlations across biomarker subgroups. The dashed vertical line indicates the pooled correlation estimate for the corresponding analysis. Blank cells in the weight columns indicate that the study was not included in that specific subgroup analysis; therefore, no weight was assigned.

NOTE: The following subgroup analyses (Section 3.7, Section 3.8 and Section 3.9) were conducted as pre-specified exploratory analyses to generate hypotheses regarding potential sources of heterogeneity. Given the small number of studies included in the quantitative synthesis (k = 6), these analyses are substantially underpowered and their results do not constitute confirmatory evidence. All interpretations of subgroup patterns should be regarded as directional and hypothesis-generating only. Subgroup analyses according to biomarker type demonstrated differences in the magnitude and consistency of correlations with CGM to the derived metrics (Figure 4). HbA1c showed a moderate pooled correlation (r = 0.65; 95% CI: 0.51–0.75), although with substantial heterogeneity across studies. GA demonstrated the strongest pooled correlation with CGM-derived metrics (r = 0.74; 95% CI: 0.65–0.81), with lower between-study dispersion. In contrast, fructosamine demonstrated more variable correlations with wider confidence intervals, and these findings should be interpreted cautiously given the limited number of included studies.

### 3.8. Heterogeneity and Subgroup Analysis (Exploratory)

Clinical and methodological heterogeneity was identified across the included studies. Potential sources of variability included differences in CKD clinical populations, dialysis modality, CGM-derived metrics, and sample size distribution. These considerations supported the use of random-effects models for quantitative synthesis.

**Figure 5:**
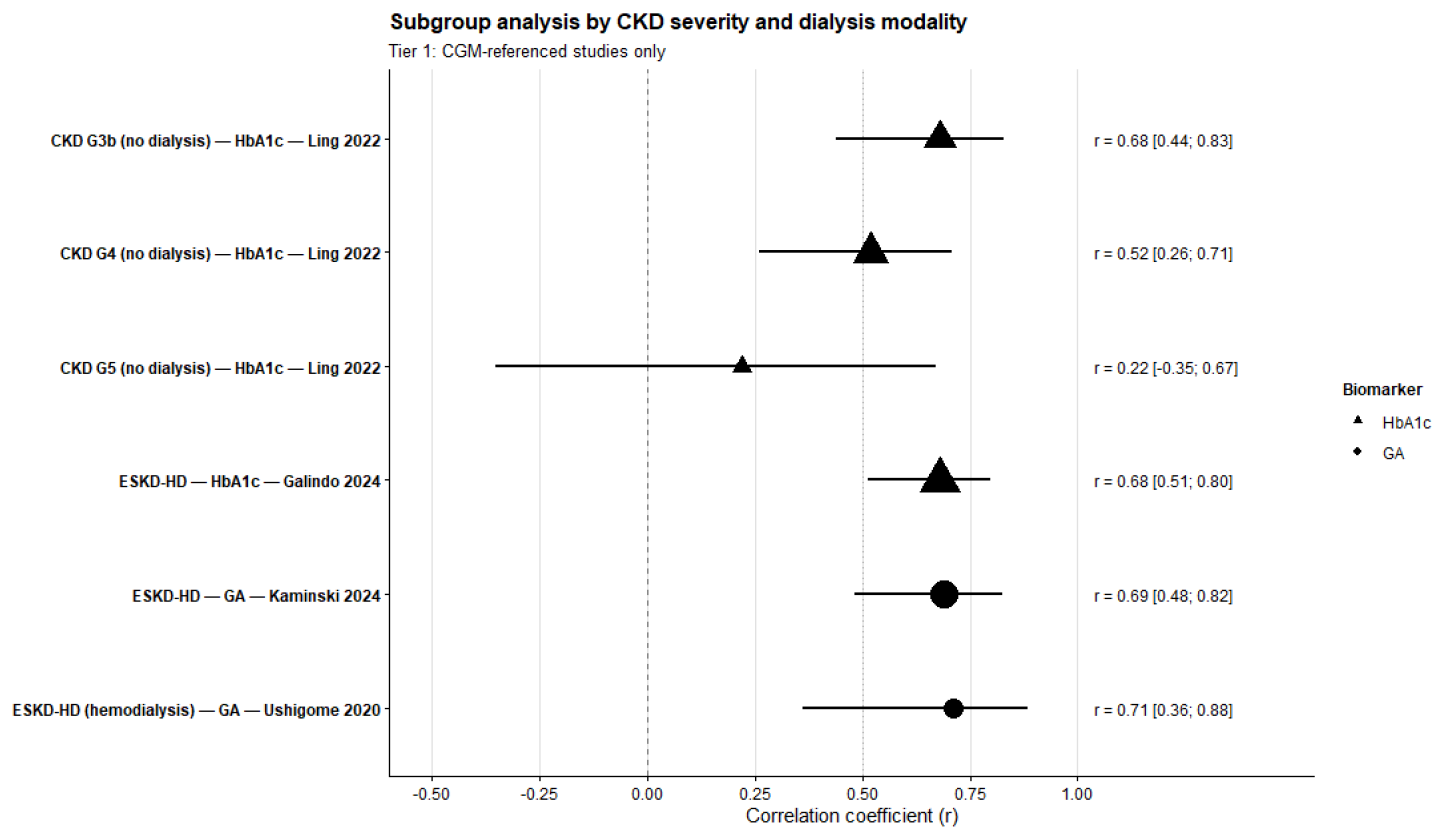
Subgroup analysis according to CKD severity and dialysis modality (Tier 1: CGM-referenced studies only). Point-range plot showing individual study correlation coefficients between glycemic biomarkers and continuous glucose monitoring-derived metrics across CKD severity categories and dialysis modalities. Horizontal lines represent 95% confidence intervals. Symbol size is proportional to study weight (inverse variance). Triangles indicate HbA1c, whereas circles indicate glycated albumin. Data from Ling et al. [8] illustrate the progressive attenuation of the correlation between HbA1c and GMI with advancing CKD stage, from G3b to G5. Data from Galindo et al. [9], Kaminski et al. [22], and Ushigome et al. [23] correspond to patients with ESKD undergoing hemodialysis.

Subgroup analysis demonstrated substantial variability in biomarker performance according to CKD severity and dialysis modality (Figure 5). Higher pooled correlations were observed in non-CKD populations and dialysis subgroups using glycated albumin, whereas lower correlations tended to be observed in advanced CKD stages, particularly among very severe CKD populations. Heterogeneity was greater in moderate and severe CKD categories, suggesting that disease severity may contribute to interstudy variability in biomarker performance.

The subgroup meta-analysis demonstrated a moderate overall correlation between glycemic biomarkers and CGM-derived metrics (r = 0.66; 95% CI: 0.56–0.74), with substantial heterogeneity (I^2^ = 58.9%). GA demonstrated the highest pooled correlation (r = 0.74; 95% CI: 0.65–0.81), although heterogeneity was low (I^2^ = 0.0%). HbA1c showed a moderate pooled correlation (r = 0.65; 95% CI: 0.51–0.75) with considerable heterogeneity (I^2^ = 74.1%). Fructosamine demonstrated lower and more variable correlations across studies.

No statistically significant differences were identified between biomarker subgroups (*p* > 0.05), suggesting that biomarker category alone did not fully explain the observed heterogeneity. These findings should be interpreted cautiously given the limited number of included studies.

Subgroup analysis demonstrated substantial variability in biomarker performance according to CKD severity and dialysis modality. Higher pooled correlations were observed in non-CKD populations and dialysis subgroups using glycated albumin, whereas lower correlations were identified in advanced CKD stages, particularly among very severe CKD populations. Heterogeneity was greater in moderate and severe CKD categories, suggesting that disease stage contributes importantly to variability in biomarker accuracy across studies.

### 3.9. Subgroup Analysis by CKD Severity (Exploratory)

**Figure 6:**
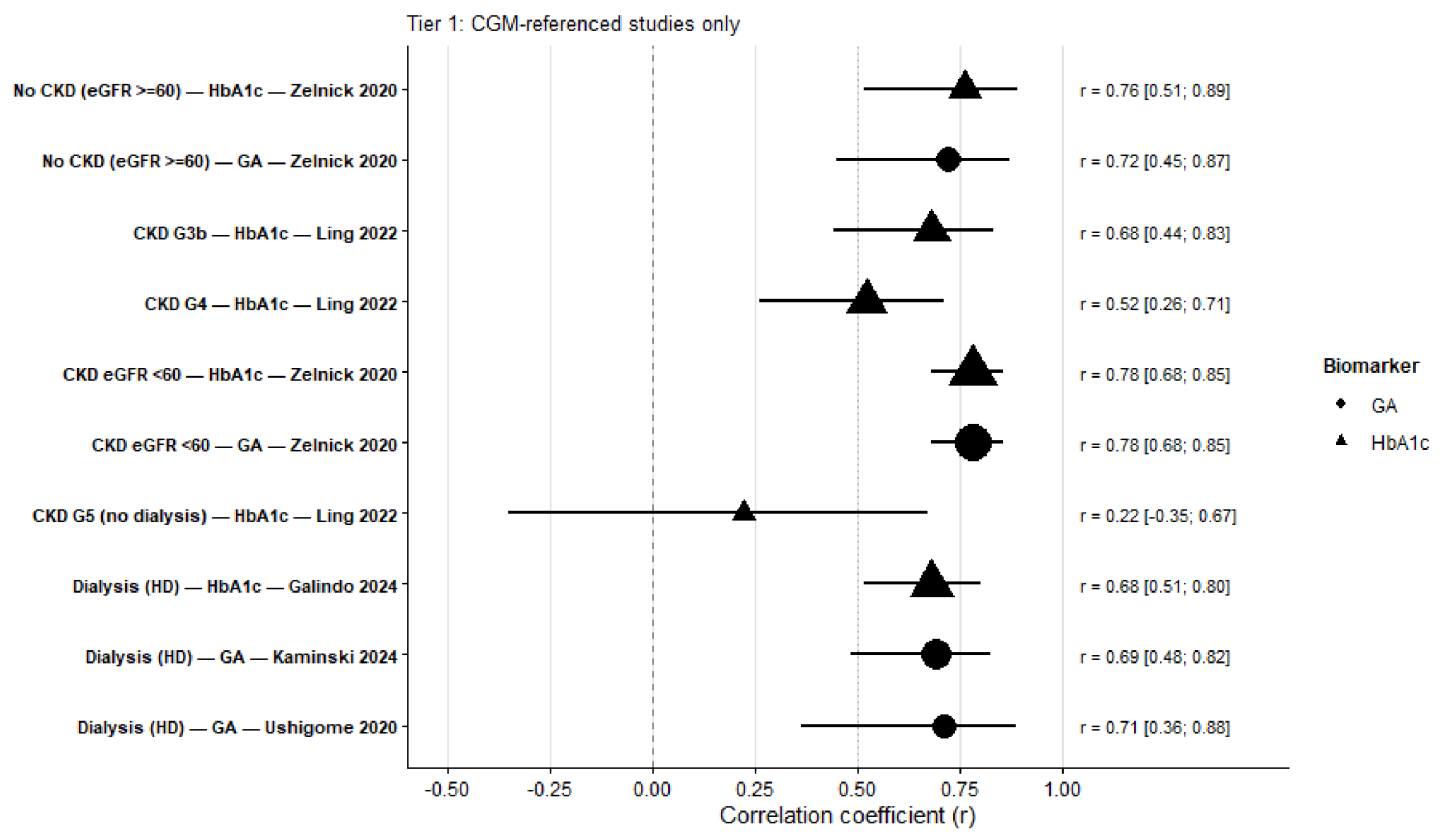
Subgroup analysis of correlations between CKD severity, glycemic biomarkers, and continuous glucose monitoring to derived metrics (Tier 1: CGM-referenced studies only) [8,9,17,22,23].

An exploratory subgroup analysis examined individual study correlations across CKD severity categories and dialysis modalities (Figure 6). Progressive attenuation of the HbA1c to CGM correlation with advancing CKD stage was observed (Ling et al. [8] (G3b r = 0.68, G4 r = 0.52, G5 r = 0.22), which demonstrates that in end-stage disease, HbA1c becomes clinically uninformative for daily management, a finding that mandates the integration of CGM or GA in dialysis units. No CKD subgroup moderator reached statistical significance (QM [df = 3] = 3.55, *p* = 0.314). None of the subgroup-specific coefficients reached statistical significance, including dialysis subgroup (*p* = 0.128), ESKD subgroup (*p* = 0.151), and mixed CKD subgroup (*p* = 0.138).

A binary moderator analysis comparing dialysis/ESKD studies (Galindo et al. [9], Kaminski et al. [22], and Ushigome et al. [23]) with non-dialysis CKD studies (Ling et al. [8], Zelnick et al. [17], and Oriot et al. [10]) did not reach statistical significance (Q_M [df = 1] = 0.289, *p* = 0.591). The moderator did not explain the residual heterogeneity (I^2^ = 65.2%, τ^2^ = 0.0278, R^2^ = 0.0%). The regression coefficients are presented in Table 2.

### 3.10. Publication Bias

Publication bias was assessed through the visual inspection of funnel plots constructed from Fisher z-transformed correlation coefficients (Supplementary Materials Figure S1). A limited number of independent estimates were available for quantitative synthesis (*k* = 6), reducing the statistical power to reliably detect funnel plot asymmetry or small-study effects. No clear asymmetry pattern was observed; however, these findings should be interpreted cautiously given the limited number of studies included.

## 4. Discussion

The objective of this systematic review and meta-analysis was to evaluate the association between HbA1c, GA, and fructosamine and CGM-derived glycemic metrics in patients with diabetes and CKD. Unlike previous narrative reviews, our quantitative synthesis identifies a critical threshold of biomarker failure [26].

Three interpretive themes emerge from integrating the individual study findings with the pooled analyses. First, CKD stage is the primary moderator of biomarker validity: Ling et al. [8] documented progressive correlation attenuation (G3b: r = 0.68; G4: r = 0.52; G5: r = 0.22), and Jung et al. [16] confirmed systematic deterioration with declining glomerular filtration rate in the ARIC cohort. This stage-dependent pattern mandates individualized biomarker interpretation rather than a uniform clinical approach. Second, dialysis modality modifies GA and HbA1c performance differently: GA shows stronger associations with CGM metrics in dialysis settings, particularly peritoneal dialysis, but its advantages are limited by susceptibility to hypoalbuminemia, obesity, and inflammatory status, which independently alter GA levels through non-glycemic mechanisms. Third, fructosamine does not demonstrate consistent added value in any identified clinical context: its dependence on the total serum protein pool, combined with contributions from globulins and lipoproteins of heterogeneous metabolic half-lives, reduces its biological specificity as a glycemic marker in CKD. In relation to the prior literature, the pooled r = 0.66 from this review was lower than the r = 0.78 reported by Zelnick et al. [17] for preserved renal function, as expected given the inclusion of advanced CKD and dialysis populations where correlation attenuation is well-documented. The absence of significant moderators (all *p* > 0.05) should not be interpreted as clinical homogeneity but as insufficient statistical power at k = 6 studies.

The results show a moderate overall pooled correlation (r = 0.66; 95% CI: 0.56–0.74) accompanied by substantial heterogeneity (I^2^ = 58.9%), indicating considerable variability across studies and limiting the interpretability of the pooled effect estimate. In this context, no biomarker demonstrated consistent superiority, precluding its use as a reliable substitute for CGM in advanced CKD [8,9]. When analyzed according to biomarker type, GA showed the strongest correlation with CGM-derived metrics (r = 0.74), followed by HbA1c (r = 0.65) and fructosamine (r = 0.64). However, this apparent hierarchy became less robust after considering specific confounding factors, particularly in the setting of advanced CKD [16,18].

**Figure 7:**
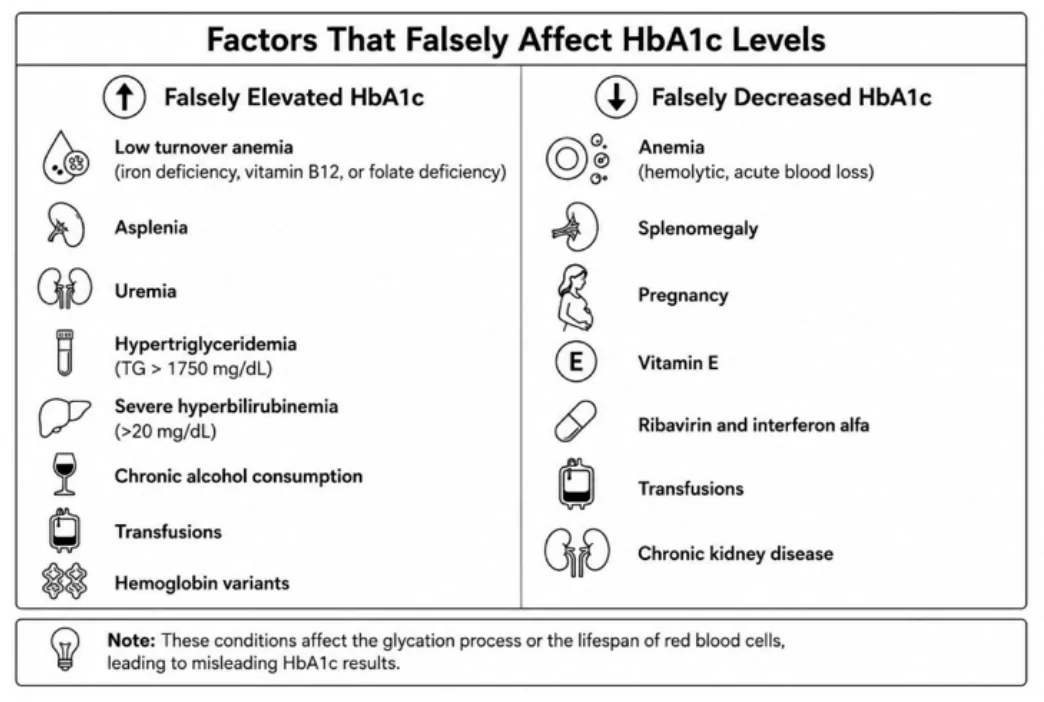
Factors that falsely affect HbA1c levels.

Consistently, HbA1c showed a marked decline in concordance as kidney disease progressed, with loss of statistical significance in CKD stage G5 (r = 0.22) [8]. This phenomenon is likely explained by structural alterations characteristic of advanced CKD, including erythrocyte lifespan, erythropoiesis-stimulating agent use, and chronic inflammation, all of which modify hemoglobin glycation independently of circulating glucose levels (Figure 7). Furthermore, a critical aspect limiting the reliability of HbA1c in routine clinical practice is the lack of universal standardization of measurement techniques. Although HbA1c is the most standardized biomarker thanks to the National Glycohemoglobin Standardization Program (NGSP), which certifies methods traceable to the Diabetes Control and Complications Trial (DCCT) reference; nevertheless, not all laboratories use high-performance liquid chromatography (HPLC), which is the reference method. Devices based on immunoassays or enzymatic methods may show clinically significant deviations, especially in the presence of hemoglobin variants, anemia, or uremia, adding an uncontrolled source of bias in many observational studies (https://ngsp.org/). Consequently, HbA1c may underestimate glycemic exposure and lose validity as an isolated biomarker in this population [9,18].

It is important to clarify that the CGM-derived metrics referred to in this meta-analysis do not constitute a single composite variable, but rather a set of physiologically relevant parameters for estimating glycemic exposure. These include mean glucose, glucose management indicator (GMI), time in range (TIR), time above range (TAR), time below range (TBR), and glycemic variability (CV). In our hierarchical analysis, mean glucose derived from CGM was prioritized as the primary reference, followed by GMI and TIR, to ensure methodological consistency across the included studies [9,18]. This distinction is relevant because each metric captures complementary aspects of glycemic control, and their heterogeneity across studies may partly explain the variability observed in the correlations.

Glycated albumin may provide a closer approximation to glycemia in specific clinical settings, particularly among patients receiving dialysis. However, its accuracy is influenced by factors such as hypoalbuminemia, inflammatory status, body mass index, and protein metabolism, limiting its general applicability [16,19,23,27,28,29]. Moreover, GA lacks a universal standardization program comparable to the NGSP. Although the International Federation of Clinical Chemistry and Laboratory Medicine (IFCC) and the Joint Committee for Traceability in Laboratory Medicine (JCTLM) have validated some methods, there is no globally accepted single reference standard, which generates variability among different commercial assays and hinders the comparison of results across centers and studies. Furthermore, GA is more expensive than HbA1c and less available in primary care clinics, particularly in healthcare systems with limited resources, which restricts its implementation as a routine alternative. In contrast, fructosamine demonstrated lower performance and greater variability, without consistent clinical advantages, as also reported by Ha et al. [15] and Zelnick et al. [17].

These findings suggest that the correlation between glycemic biomarkers and CGM-derived metrics depends largely on CKD stage and the individual clinical context, requiring careful contextual interpretation. In this setting, CGM provides the most direct real-time assessment of glycemic exposure currently available because it captures glycemic variability and intraday glucose fluctuations not reflected in traditional biomarkers; however, its accuracy in advanced CKD remains subject to the limitations described above [9,10,30,31].

Furthermore, CGM allows for the assessment of time in range (TIR), a metric with well-defined clinical objectives in the diabetic population that is strongly correlated with the risk of microvascular complications. In the specific context of CKD, CGM’s ability to provide TIR data offers a significant clinical advantage, as it avoids interference from impaired red blood cell turnover and uremic environments that typically distort HbA1c readings, enabling more precise and individualized therapeutic adjustment. Unlike glycated hemoglobin (A1c), which reflects an average over the past three months, time in range (TIR) provides a dynamic assessment of daily glycemic control. This facilitates the early identification and correction of glucose fluctuations related to meals or exercise, revealing episodes of hypoglycemia and hyperglycemia that A1c alone might miss. Maintaining a high TIR is directly associated with a significant reduction in the risk of developing diabetes-related complications.

Despite its advantages for direct glycemic measurement, CGM is not without limitations in patients with advanced CKD and dialysis. Uremia may alter interstitial fluid composition, potentially affecting the accuracy of enzymatic glucose biosensors. During hemodialysis sessions, acute fluid shifts can create transient discrepancies between interstitial and plasma glucose that do not reflect true glycemic changes. Device variability is also relevant: the included studies used four different CGM systems (Dexcom G6 Pro in Galindo 2024 [9] and Kaminski 2024 [22]; Medtronic iPro2 in Ling 2022 [8]; FreeStyle Libre in Ushigome 2020 [23]; isCGM in Oriot 2022 [10]), each with different sensor technologies, wear durations (6–90 days), and calibration requirements. Factory-calibrated and user-calibrated sensors differ in accuracy profiles, and no head-to-head comparison of these devices in advanced CKD populations is currently available. CGM is used here as the reference standard because it provides the most direct real-time measurement of interstitial glucose currently available in clinical practice, not because it constitutes an absolute gold standard free of error in this population.

Pathophysiological Explanation: The progressive divergence between biomarkers and true glycemia in patients with advanced CKD is driven by multiple pathophysiological mechanisms that affect each biomarker differently [27].

Reduced erythrocyte lifespan represents one of the principal mechanisms affecting HbA1c interpretation in CKD. In CKD, erythrocytes exhibit shortened survival due to uremia, dialysis-related factors, chronic inflammation, and erythropoietin deficiency. Because erythrocytes are exposed to circulating glucose for a shorter period, hemoglobin glycation decreases independently of actual glycemia. This mechanism may explain why HbA1c systematically underestimates glycemic exposure in advanced CKD [10,16,20,22,32].

Serum albumin concentration is a critical determinant of GA, as this biomarker is expressed as the ratio of GA to total albumin. In patients with hypoalbuminemia (<3.5 g/dL), a common condition in advanced CKD and among individuals receiving dialysis, GA levels may show upward or downward bias depending on the balance between albumin synthesis, catabolism, and loss [17].

In addition, nutritional status and body mass index substantially influence GA. Lower GA levels have been consistently observed in patients with overweight and obesity, independent of glycemia, introducing potential confounding, particularly in populations with high obesity prevalence such as those evaluated by George et al. [25] and Zelnick et al. [17].

Approximately 90% of fructosamine derives from GA; therefore, factors influencing GA similarly affect fructosamine levels. The remaining fraction originates from other serum proteins such as globulins and lipoproteins, which present longer half-lives and greater metabolic heterogeneity. This composition explains the higher variability and inferior performance of fructosamine compared with GA [15,18].

**Figure 8:**
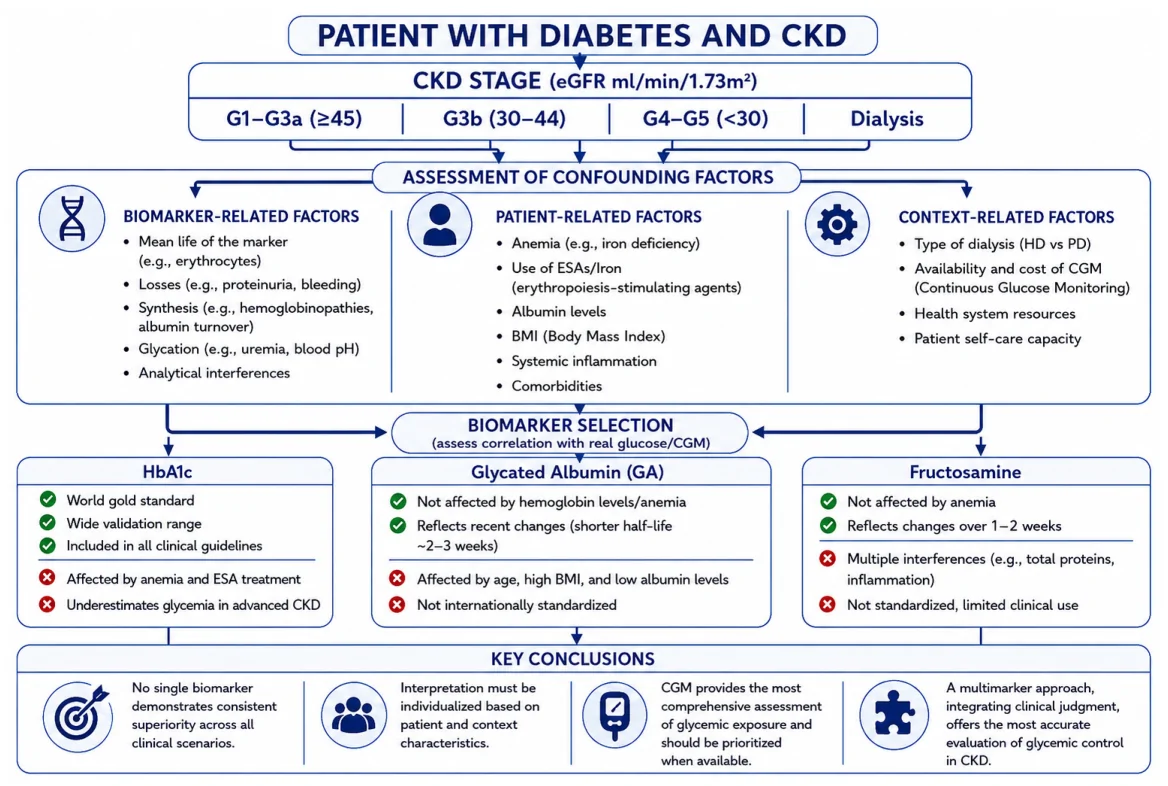
Proposed practical flowchart for contextual interpretation of glycemic biomarkers in patients with diabetes and chronic kidney disease. The flowchart summarizes a clinically contextualized approach for interpreting HbA1c, glycated albumin, and fructosamine according to CKD stage, dialysis status, anemia, nutritional condition, and availability of continuous glucose monitoring.

Clinical Implications for Practice: Based on the findings of this systematic review and meta-analysis, a practical flowchart for the contextual interpretation of glycemic biomarkers in patients with diabetes and chronic kidney disease is proposed (Figure 8).

The interpretation of glycemic biomarkers in CKD should be contextualized according to kidney disease stage, dialysis status, anemia, nutritional condition, inflammatory state, and availability of CGM.

In advanced CKD, particularly in stages G4–G5 and patients undergoing dialysis, HbA1c alone may be insufficient to accurately estimate glycemic exposure due to alterations in erythrocyte lifespan, erythropoietin use, anemia, and changes in glycation kinetics associated with uremia and chronic inflammation [4,28,33,34]. This limitation becomes progressively more evident as renal function declines and contributes to discordance between HbA1c and CGM-derived metrics.

GA may provide a useful complementary assessment in advanced CKD and dialysis settings, especially when CGM is unavailable [27,28,35]. Nevertheless, its interpretation remains susceptible to non-glycemic influences, including serum albumin concentration, protein metabolism, body mass index, and inflammatory status [29,35,36,37].

CGM appears to provide the most physiologically robust estimation of glycemic exposure in advanced CKD because it captures mean glucose levels, glycemic variability, and temporal glucose fluctuations that are not adequately reflected by conventional biomarkers [6,23,32]. In this context, continuous glucose monitoring may facilitate a more comprehensive characterization of glycemic patterns in patients with advanced renal dysfunction.

In contrast, fructosamine showed greater variability across studies and less consistent correlations with glycemic reference metrics, particularly across different CKD stages and dialysis settings [15,27,38]. This variability may be related to its dependence on circulating serum proteins with heterogeneous half-lives and to the influence of non-glycemic factors affecting protein metabolism [29]. Consequently, its role as a primary biomarker for glycemic assessment in chronic kidney disease has not been consistently established.

Overall, the findings suggest that no single biomarker demonstrates consistent superiority across all clinical scenarios. Therefore, the interpretation of HbA1c, GA, and fructosamine should be individualized according to CKD severity, dialysis modality, anemia status, nutritional condition, and access to CGM technologies.

These findings are consistent with evidence from the studies with the highest methodological quality included in this review and support a progressive decline in biomarker validity as CKD advances.

Ling et al. documented a quantitative reduction in the correlation between HbA1c and GMI, with coefficients decreasing from r = 0.68 in CKD stage G3b to r = 0.22 in stage G5 [8]. Jung et al. confirmed this pattern, demonstrating progressive deterioration in correlation as glomerular filtration rate declines [16]. Together, these findings suggest substantial loss of discriminative capacity in advanced CKD.

George et al. [25] showed that loss of diagnostic validity does not follow a uniform pattern and appears to intensify at higher glycemic levels; they identified an underestimation of true glycemia among patients with advanced CKD and elevated HbA1c values, with a significant interaction effect (*p* = 0.030). In contrast, GA did not show a consistent interaction effect (*p* = 0.95), contributing to heterogeneity in clinical interpretation.

Despite this progressive deterioration, HbA1c appears to maintain better overall performance in patients with preserved or mildly impaired kidney function. Using CGM as the reference standard, Zelnick et al. [17] reported greater accuracy for HbA1c (p10 = 77%) compared with GA (56%) and fructosamine (61%) [17].

Similarly, George et al. demonstrated stronger correlations between HbA1c and fasting glucose (r = 0.59) compared with GA (r = 0.44) and fructosamine (r = 0.52) in the general CKD population, supporting its relative utility in earlier disease stages [25].

GA may provide additional value in selected clinical scenarios, particularly among patients receiving dialysis therapy and those with “burnt-out diabetes”. Galindo et al. [9] demonstrated that in hemodialysis populations, the glucose management indicator showed a stronger correlation with time in range (r = −0.96) compared with laboratory HbA1c (r = −0.67). Furthermore, 49% of patients showed discordance greater than 1% between HbA1c and GMI.

This review has several limitations that affect its internal and external validity. (1) Heterogeneity and small k: The quantitative synthesis included k = 6 studies, severely limiting the statistical power of subgroup and meta-regression analyses; the I^2^ of 58.9% reflects true clinical heterogeneity that cannot be decomposed with the available data, and all subgroup findings must be regarded as hypothesis-generating. (2) Correlation as effect measure: Correlation quantifies linear association, not agreement or interchangeability; Bland–Altman limits of agreement and intraclass correlation coefficients, the appropriate agreement metrics, cannot be derived from aggregate study-level data, limiting clinical translation of the findings. (3) CGM device variability (internal validity): Four distinct CGM systems with different calibration requirements were used across studies; interstitial glucose sensing may be differentially affected by uremia and fluid shifts across devices, introducing a source of measurement bias that cannot be adjusted without individual patient data. (4) Biomarker assay heterogeneity (internal validity): Variability in HbA1c assay methods and the absence of universal GA standardization contribute to between-study measurement differences. (5) External validity: The predominance of high-income settings with CGM availability limits generalizability to healthcare systems with restricted CGM access and to peritoneal dialysis populations. (6) Observational design and residual confounding: All included studies are observational; residual confounding from anemia severity, erythropoiesis-stimulating agent use, nutritional status, and inflammatory markers cannot be controlled at the meta-analytic level. (7) Absence of clinical outcome data: No included study evaluated whether biomarker-CGM concordance predicts hypoglycemia, cardiovascular events, or mortality; this constitutes the critical priority for future prospective research.

Finally, the predominance of studies with low-to-moderate risk of bias and the concentration of studies in settings with high availability of CGM may limit the generalizability of these findings to healthcare environments with more restricted access to this technology.

An additional conceptual limitation concerns the use of correlation coefficients as the primary effect measure. Correlation quantifies the strength and direction of linear association between two variables, but does not measure agreement or clinical interchangeability. A high correlation does not imply that two measurements are numerically similar or that one can substitute the other in clinical decision-making. Proper quantification of agreement would require individual patient data and methods such as Bland–Altman limits of agreement or intraclass correlation coefficients, which cannot be derived from the summary-level statistics available in this review. Consequently, none of the findings support conclusions about the diagnostic substitutability of any biomarker for another, a point explicitly acknowledged throughout the interpretation of results.

A further limitation is the exclusive focus on biomarker–CGM correlation analyses, without an evaluation of associations with clinically meaningful outcomes. Available prognostic data were insufficient for formal quantitative synthesis: Bulatovic et al. (2026) [12] reported an independent association between elevated glycated albumin and cardiovascular mortality in CKD stage V patients over five years, and Mayeda et al. (2020) [18] demonstrated that higher CGM-derived time in range was associated with reduced peripheral neuropathy risk in CKD populations. Establishing whether any glycemic biomarker is superior for predicting hypoglycemia, major cardiovascular events, or mortality in CKD constitutes a critical priority for future prospective research with standardized CGM protocols and individual patient-level outcome data.

Additional limitations include the observational design of all included studies, variability in CGM devices and monitoring duration, differences in biomarker assays, possible residual confounding due to anemia and nutritional status, limited availability of individual participant data, and the small number of studies available for subgroup analyses. These limitations may reduce both the internal validity and generalizability of the pooled estimates.

Nevertheless, the consistency of the observed patterns across studies supports the validity of the clinical relevance of the main findings in advanced CKD populations.

## 5. Conclusions

Biomarker validity in CKD is not a static property but a stage-dependent continuum. In early CKD, HbA1c retains reasonable correlation with CGM-derived metrics and remains a practical first-line tool. As CKD advances beyond stage G4 and in dialysis populations, CGM is the only reference capable of reliably capturing glycemic exposure; where unavailable, GA may provide complementary information provided non-glycemic confounders are considered. No biomarker demonstrated consistent superiority across all evaluated CKD stages and treatment modalities, and none could substitute for another in clinical practice based on correlation alone. Future research must prioritize outcome-based trials with harmonized CGM protocols to determine whether biomarker selection reduces hypoglycemia, cardiovascular events, or CKD progression.

## Figures and Tables

**Table 1:** Characteristics of the studies included in the systematic review.

Author/Year/ Country	Design and Scope	Population/Sample	Evaluated Biomarkers	Reference/Comparator	Statistical Analysis	Relevant Main Finding
Galindo et al., [9] 2024/USA	Prospective observational study	59 adults with T2DM and CKD undergoing hemodialysis treatment	HbA1c CGMI metrics: Average glucose TIR CV	CGM Dexcom G6 Pro, correlation analysis with average glucose, GMI, and TIR	GMI-TIR: r = 0.96 GMI-HbA1c: r = 0.68 HbA1c-average glucose: r = 0.68 Discrepancy > 1% between HbA1c and GMI: 49% of participants.	HbA1c may underestimate or overestimate glycemic control in hemodialysis patients; CGM metrics provide a more accurate assessment of actual glycemic control.
George et al. [25], 2020/South Africa	Cross-sectional and analytical observational study	1621 adults; 1525 without CKD and 96 with CKD in stages G3, G4, and G5, without dialysis treatment.	HbA1c Glycated albumin Fructosamine	Fasting glucose	FPG and HbA1c: *p* = 0.030 FPG and GA: *p* = 0.095 FPG and Fructosamine: *p* = 0.851	HbA1c and GA showed lower concordance with fasting glucose in patients with CKD, underestimating glycemia at elevated ranges; fructosamine did not show interaction with CKD.
Jung et al., [16] 2018/USA	Cross-sectional observational and analytical study of a population cohort	1665 elderly individuals with T2DM	HbA1c Glycated albumin Fructosamine	Fasting glucose	Severe CKD: HbA1c r = 0.52, Glycated albumin r = 0.39, Fructosamine r = 0.41. Very severe CKD: HbA1c r = 0.48, Glycated Albumin r = 0.36, Fructosamine r = 0.36.	In severe and very severe CKD, the three biomarkers showed lower correlation with fasting glucose; no clear advantage of GA or fructosamine over HbA1c was observed.
Kaminski et al. [22], 2024/USA	Observational prospective study	40 patients on hemodialysis: 20 with CKD G5 and “burnt-out diabetes” and 20 without CKD	HbA1c Fructosamine Glycated albumin CGMI metrics: Average glucose TIR TAR	CGM Dexcom G6 Pro, correlation analysis with average glucose, TAR, TIR, and TBR	Burn-out diabetes vs. No diabetes: TIR 70–180: 80.4% vs. 94.0% (*p* < 0.001) TAR > 180: 17.2% vs. 4.6% (*p* < 0.001) Mean CGM Glucose: 141.7 vs. 125.7 mg/dL (*p* = 0.009) Glycated albumin: 17.1% vs. 14.5% (*p* = 0.022) HbA1c: 5.5% vs. 5.3% (*p* = 0.22) Fructosamine: 330.7 vs. 315.1 (*p* = 0.69)	CGM detected hyperglycemia not recognized by HbA1c; GA and CGM evaluated glycemic control better than HbA1c and fructosamine in ESKD. In patients with burn-out diabetes and CKD, HbA1c underestimates glycemic control; alternative biomarkers allow for a more accurate assessment of glycemic control.
Kobayashi et al., [11] 2016/Japan	Retrospective and cross-sectional observational study	20 diabetic patients on HD and 20 on PD, matched by age, sex, and baseline postprandial glucose	HbA1c Glycated albumin	Postprandial plasma glucose Levels of AG and HbA1c	GA (%): PD 17.8 ± 2.5 vs. HD 20.8 ± 2.8 (*p* < 0.001) HbA1c: PD 6.0 ± 0.6 vs. HD 6.1 ± 0.7 (*p* = 0.95, NS) Multiple regression: GA was the only independent predictor of glycemia in PD (β = 11.8, *p* = 0.007)	Glucose correlated with GA in both groups, but not with HbA1c; GA was the only independent factor associated with glucose in patients on peritoneal dialysis. In peritoneal dialysis, glycated albumin is a better indicator than HbA1c.
Ling et al. [8], 2022/China	Cross-sectional observational prospective study	90 patients with T2DM and CKD G3b to G5 without dialysis treatment	HbA1c CGMI metrics: Average glucose TIR CV	Dexcom G6, correlation analysis with: Average glucose, GMI, TIR, Time in hyperglycemia, Time in hypoglycemia, and CV	GMI-HbA1c: G3b: r = 0.68 (*p* < 0.0001) G4: r = 0.52 (*p* < 0.001) G5: r = 0.22 (*p* = 0.44) HbA1c-time in hypoglycemia: r = −0.045	The HbA1c to GMI correlation decreased with the progression of CKD; in eGFR < 30 mL/min/1.73 m^2^, HbA1c is unreliable, does not reflect true hyperglycemia, and does not predict the risk of hypoglycemia.
Ha et al., [15] 2024/Vietnam	Hospital case–control study	100 participants (50 cases with CKD and 50 controls without CKD), with T2DM, with and without CKD	HbA1c Fructosamine Fructosamine/albumin ratio	Fasting glucose	HbA1c to fasting glucose: Without CKD: r = 0.758 With CKD: r = 0.575 Fructosamine, fasting blood glucose in CKD: r = 0.231 AUC for HbA1c in CKD: 0.733 AUC for fructosamine in CKD stage 4: 0.844	HbA1c remains useful in CKD, but in advanced stages (eGFR < 30), fructosamine and the fructosamine/albumin ratio can be alternatives for short-term monitoring.
Oriot et al. [10], 2022/Belgium, France	Multicenter retrospective and observational study	355 patients with T2DM: 170 with CKD 185 without CKD	HbA1c Hemoglobin Hematocrit Iron status	CGM 14 and 90-day GMI	Difference HbA1c, GMI: CKD 0.78 ± 0.57% vs. without CKD 0.59 ± 0.44% (*p* = 0.005) Discrepancy > 0.5%: 68.2% in CKD vs. 42.2% in non CKD	The HbA1c to GMI discordance was greater in CKD than in non-CKD, indicating inappropriate treatment adjustments and risk of hypoglycemia.
Ushigome et al. [23], 2020/Japan	Analytical, observational, and cross-sectional study	118 patients with T2DM undergoing hemodialysis	Glycated albumin eHbA1cGA eHbA1c Serum hemoglobin Average interstitial glucose Serum albumin	FreeStyle 14-Day Flash Glucose Monitoring System	Difference eHbA1c(GA), eHbA1c(CGM): BMI < 18.5: 3.3 ± 0.6% BMI ≥ 18.5: 1.6 ± 1.2% (*p* = 0.005)	Estimated hemoglobin A1c using FGM is a more reliable indicator of glycemic control than the estimation based on glycated albumin in patients with low BMI and/or on hemodialysis.
Zelnick et al. [17], 2020/USA	Prospective observational cohort study	104 patients with T2DM: 80 with CKD 24 without CKD	HbA1c Glycated albumin Fructosamine	CGM Average CGM glucose	Blood sugar average CGM: HbA1c r = 0.78; GA r = 0.77; fructosamine r = 0.71	The three biomarkers correlated similarly with mean CGM glucose (r = 0.71–0.78), but HbA1c showed lower overall variability.

HbA1c: glycated hemoglobin, GA: glycated albumin, FPG: Fasting plasma glucose, eGFR: estimated glomerular filtration rate, CKD: chronic kidney disease, TDM: type 2 diabetes, CGM: continuous glucose monitoring, GMI: glucose management indicator, TBR: time below range, TIR: time in range, TAR: time above range, CV: glycemic variability, isCGM: intermittent monitoring, eHbA1c: continuous monitor-based hemoglobin A1c (FGM), eHbA1cGA: estimated hemoglobin A1c based on GA.

**Table 2:** Exploratory subgroup analysis: regression coefficients using Fisher z-transformed effect sizes (Tier 1: CGM-referenced studies only, k = 6).

Parameter	Estimate (z)	SE	z Value	*p* Value	95% CI
Model 1: Dialysis moderator					
Intercept	0.7514	0.1121	6.705	<0.001	0.5318–0.9711
Dialysis subgroup	0.0966	0.1796	0.538	0.591	−0.2555–0.4486
Model 2: CKD severity moderator					
Intercept	0.8480	0.1404	6.041	<0.001	0.5729–1.1231
Non-dialysis CKD	−0.0966	0.1796	−0.538	0.591	−0.4486–0.2555

Two separate models were fitted due to collinearity among subgroup moderators with k = 6. Model 1 contrasts dialysis vs. non-dialysis studies. Model 2 contrasts non-dialysis CKD vs. ESKD/dialysis. Both models: QM (df = 1) = 0.289, *p* = 0.591; I^2^ = 65.2%, τ^2^ = 0.0278, R^2^ = 0.0%. No moderator reached statistical significance. These analyses are exploratory and substantially underpowered; results should not be interpreted as confirmatory evidence.

## Data Availability

The data supporting the findings of this study are available from the corresponding author upon reasonable request. The extracted dataset and analysis files can be provided for academic and research purposes, subject to applicable ethical and institutional requirements.

## Supplementary Materials

The following supporting information can be downloaded at: https://www.mdpi.com/article/10.3390/jcm15166137/s1, Table S1: Search strategy, Table S2: QUADAS-2. Risk of Bias Assessment (Tier 1: CGM-referenced studies only) Note: George et al., [25], Jung et al., [16], Kobayashi et al., [11], and Ha et al., [15] (Tier 2) were assessed using NOS only and are not included in this table, Table S3: Methodological Quality Assessment (Newcastle-Ottawa Scale, NOS). All 10 included studies assessed. Studies in Tier 2 [11,15,16,25], used fasting glucose as comparator and are included in narrative synthesis only. Figure S1: Funnel Plot of Effect Sizes in the Meta-analysis of the Correlation Between Glycemic Biomarkers and Metrics Derived from Continuous Glucose Monitoring. Tier 1: CGM-referenced studies only (k = 6). Each point represents a study according to its Fisher z-transformed correlation and standard error. The dashed line indicates the pooled effect (z = 0.787; r = 0.66). The triangular area represents the expected 95% CI distribution in the absence of publication bias. Visual inspection suggests no marked asymmetry; however, formal Egger test is not reliable with fewer than 10 studies and results should be interpreted with caution. Supplementary S1. PRISMA Checklist [19].

## Abbreviations

The following abbreviations are used in this manuscript:

HbA1c	Glycated Hemoglobin
AG	Glycated Albumin
AUC	Area Under the Curve
BMI	Body Mass Index
CGM	Continuous Glucose Monitoring
CI	Confidence Interval
CKD	Chronic Kidney Disease
CV	Coefficient of Variation
eGFR	Estimated Glomerular Filtration Rate
eHbA1c	Estimated Hemoglobin A1c
eHbA1c (GA)	Estimated Glycated Hemoglobin Based on Glycated Albumin
ESKD	End-Stage Kidney Disease
FGM	Flash Glucose Monitoring
FPG	Fasting Plasma Glucose
GMI	Glucose Management Indicator
HD	Hemodialysis
I^2^	Statistical Heterogeneity Index
MeSH	Medical Subject Headings
PD	Peritoneal Dialysis
PRISMA	Preferred Reporting Items for Systematic Reviews and Meta-Analyses
PROSPERO	International Prospective Register of Systematic Reviews
QM	Q statistic of The Meta-Regression or Subgroup Analysis Model
QUADAS-2	Quality Assessment of Diagnostic Accuracy Studies-2
r	Correlation Coefficient
TAR	Time Above Range
TBR	Time Below Range
TIR	Time In Range
T2DM	Type 2 Diabetes
USA	United States of America
vs.	Versus
