# Supplementary material for: Correlation Between Glycemic Biomarkers and Continuous Glucose Monitoring Metrics in Chronic Kidney Disease: A Systematic Review and Correlation Meta-Analysis
Source: J Clin Med. 2026 Aug 7;15(16):6137. doi: 10.3390/jcm15166137 (PMC13513047; doi:10.3390/jcm15166137)
Supplement: Supplementary file 1 [file jcm-15-06137-s001.zip › jcm-4403743-supplementary.pdf]

Supplementary Table S1. Search Strategy.

Data base	Proposed Literature Search Strategy
PubMed	(("Hemoglobin A, Glycosylated"[Mesh] OR HbA1c[tiab] OR "glycated hemoglobin"[tiab] OR "glycosylated hemoglobin"[tiab] OR "glycated albumin"[tiab] OR fructosamine[tiab]) AND ("Renal Insufficiency, Chronic"[Mesh] OR "Kidney Failure, Chronic"[Mesh] OR "chronic kidney disease"[tiab] OR CKD[tiab] OR "renal failure"[tiab] OR "renal insufficiency"[tiab] OR dialysis[tiab]) AND ("glycemic control"[tiab] OR "metabolic control"[tiab] OR accuracy[tiab] OR validity[tiab] OR reliability[tiab] OR correlation[tiab] OR "continuous glucose monitoring"[tiab] OR CGM[tiab])) AND ("2016/01/01"[Date - Publication] : "2025/03/31"[Date - Publication]
Scopus	TITLE-ABS-KEY ((HbA1c OR "glycated hemoglobin" OR "glycosylated hemoglobin" OR "glycated albumin" OR fructosamine) AND ("chronic kidney disease" OR CKD OR "renal failure" OR "renal insufficiency" OR dialysis) AND ("glycemic control" OR "metabolic control" OR accuracy OR validity OR reliability OR correlation OR "continuous glucose monitoring" OR CGM))
Web of Science Core Collection	(HbA1c OR "glycated hemoglobin" OR "glycosylated hemoglobin" OR "glycated albumin" OR fructosamine) AND ("chronic kidney disease" OR CKD OR "renal failure" OR "renal insufficiency" OR dialysis) AND ("glycemic control" OR "metabolic control" OR accuracy OR validity OR reliability OR correlation OR "continuous glucose monitoring" OR CGM)

Supplementary Table S2. QUADAS-2. Risk of Bias Assessment (Tier 1: CGM-referenced studies only)
Note: George et al., [25], Jung et al., [16], Kobayashi et al., [11], and Ha et al., [15] (Tier 2) were assessed using NOS only and are not included in this table.

Study	Patient Selection	Index Test	Reference Standard	Flow and Timing	Overall Risk of Bias
Galindo 2024	Some concerns	Unclear	Low	Low	Some concerns
Ling 2022	Low	Low	Low	Low	Low
Zelnick 2020	Low	Low	Low	Low	Low
Kaminski 2024	Some concerns	Unclear	Low	Low	Some concerns
Oriot 2023	Some concerns	Low	Low	Low	Some concerns
Ushigome 2020	Some concerns	Unclear	Some concerns	Low	Some concerns

Note: QUADAS-2 applied to Tier 1 studies (CGM-referenced) only. Tier 2 studies (George 2020, Jung 2018, Kobayashi 2016, Ha 2024) assessed with NOS.

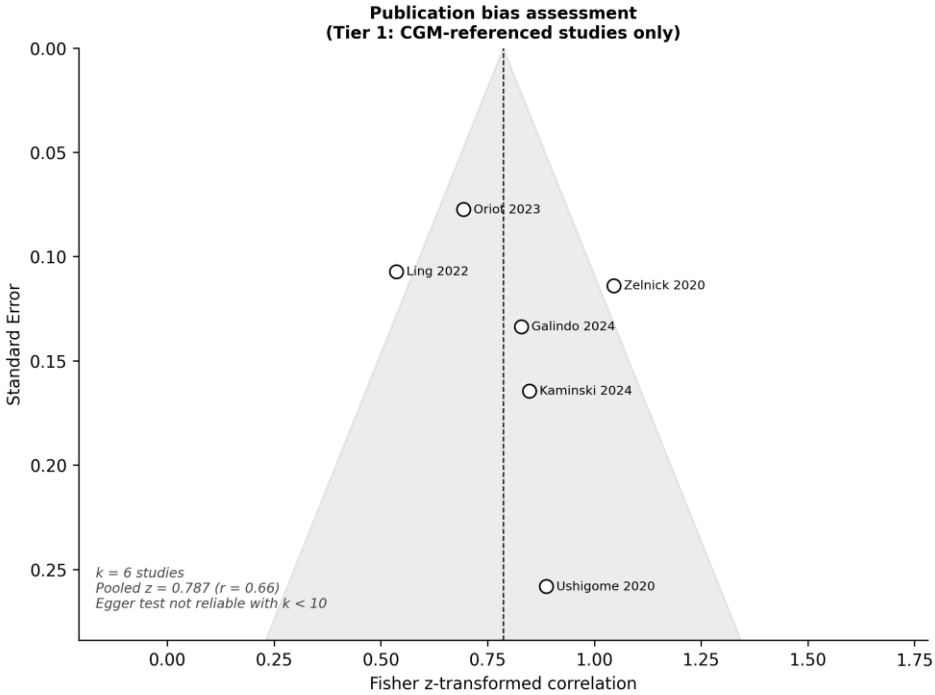

Supplementary Figure S1. Funnel Plot of Effect Sizes in the Meta-analysis of the Correlation Between Glycemic Biomarkers and Metrics Derived from Continuous Glucose Monitoring. Tier 1: CGM-referenced studies only (k = 6). Each point represents a study according to its Fisher z-transformed correlation and standard error. The dashed line indicates the pooled effect (z = 0.787; r = 0.66). The triangular area represents the expected 95% CI distribution in the absence of publication bias. Visual inspection suggests no marked asymmetry; however, formal Egger test is not reliable with fewer than 10 studies and results should be interpreted with caution.

Supplementary Table S3. Methodological Quality Assessment (Newcastle-Ottawa Scale, NOS). All 10 included studies assessed. Studies in Tier 2 [11,15,16,25], used fasting glucose as comparator and are included in narrative synthesis only.

Study	Design	Sample size	Sample selection	Biomarker measurement	Glycemic reference	Confounding control	Overall risk
Galindo 2024	Prospective observational	59	Adequate (defined criteria HD)	Standardized (HbA1c + CGM)	CGM (robust)	Limited	Moderate
Ling 2022	Observational	90	Adequate (stratified by CKD)	Standardized	CGM	Partial (eGFR considered)	Moderate
Zelnick 2020	Prospective cohort	104	Adequate (CKD + controls)	Standardized	CGM (duplicate measurement)	Adjusted (age, BMI, etc.)	Low-moderate
George 2020	Cross-sectional	1621	Community-based (heterogeneous)	Standardized	Fasting glucose	Adjusted	Moderate
Jung 2018	Cross-sectional	1665	ARIC cohort (well-defined)	Standardized	Fasting glucose	Adjusted	Moderate
Kobayashi 2016	Observational	40	Limited (HD vs PD)	Standardized	Fasting glucose	Limited	High
Ha 2024	Case control	50	Hospital-based	Standardized	Fasting glucose	Partial	Moderate
Kaminski 2024	Prospective pilot	40	Highly specific (burnt-out DM)	Standardized + CGM	CGM	Limited	High
Oriot 2023	Multicenter observational	355	Adequate	Standardized	CGM (GMI)	Partial	Moderate
Ushigome 2020	Observational	18	Very limited	Standardized	Derived CGM	Limited	High
